# Particle Swarm Inspired Underwater Sensor Self-Deployment

**DOI:** 10.3390/s140815262

**Published:** 2014-08-19

**Authors:** Huazheng Du, Na Xia, Rong Zheng

**Affiliations:** 1 School of Computer and Information, Hefei University of Technology, Hefei 230009, China; E-Mail: huazheng2616@163.com; 2 Department of Computing and Software, McMaster University, Hamilton L8S4K1, Canada; E-Mail: rzheng@mcmaster.ca

**Keywords:** underwater sensor networks, sensor deployment, coverage efficiency, particle swarm, crowd factor, water flow field

## Abstract

Underwater sensor networks (UWSNs) can be applied in sea resource reconnaissance, pollution monitoring and assistant navigation, *etc.*, and have become a hot research field in wireless sensor networks. In open and complicated underwater environments, targets (events) tend to be highly dynamic and uncertain. It is important to deploy sensors to cover potential events in an optimal manner. In this paper, the underwater sensor deployment problem and its performance evaluation metrics are introduced. Furthermore, a particle swarm inspired sensor self-deployment algorithm is presented. By simulating the flying behavior of particles and introducing crowd control, the proposed algorithm can drive sensors to cover almost all the events, and make the distribution of sensors match that of events. Through extensive simulations, we demonstrate that it can solve the underwater sensor deployment problem effectively, with fast convergence rate, and amiable to distributed implementation.

## 1. Introduction

Underwater Sensor Networks (UWSNs) are underwater monitoring network systems consisting of sensor nodes with computing and acoustic communication abilities. Due to their important applications in sea resource reconnaissance, pollution monitoring and navigation assistance, UWSNs have attracted much attention from government agencies and research institutions, and have become a hot area in sensor network research [1–3]. In recent years, many aspects of UWSNs such as underwater communication [4,5], sensor deployment and self-organization [6,7], data routing [8,9], and localization and tracking [10–12] have been investigated. Among these, sensor deployment is a key research topic since it not only determines the monitoring quality of the networks, but also acts as the foundation of the network organization, protocol design and application deployment.

Existing underwater sensor deployment methods fall into three categories: Sea-bottom, Sea-surface and Sea-column sensor deployment briefly discussed as follows:

(1) *Sea-bottom sensor deployment*. In these methods, sensors are anchored to the sea bottom to form a two-dimensional monitoring network. The monitoring area is divided into triangle or square grids in which sensors are deployed. The goal is to use as few sensors as possible to cover the monitoring area [6,13]. Sea-bottom sensor deployment is similar to land sensor placement with regards to research objectives and methods, and does not truly reflect the characteristics of UWSNs. In most applications, we need to collect the three-dimensional information of an underwater environment. As a result, sea-bottom sensor deployment is insufficient.
(2) *Sea-surface* *sensor* *deployment*. In this category, sensors (gateways or data collectors) are deployed on the sea surface, and collect data from underwater sensor nodes. Sea-surface sensor deployment is generally formulated as an optimization problem. In [14–19], the positions of sensors are computed by an Integer Linear Program (ILP), greedy algorithm and aided geometrical algorithm, so that the number of sensors can be reduced, and the network lifetime can be prolonged. But this line of work mostly still assumes two-dimensional sensor deployment.
(3) *Sea-column sensor deployment*. In this category, sensors are deployed in the three-dimensional underwater space. Existing sea-column sensor deployment methods can be further divided into two classes:

The first class is *uniform coverage requirement sensor deployment*. Sensors are uniformly deployed in the monitoring space. In 2006, Pompili *et al.* [6,7] pioneered the research in three-dimensional underwater sensor deployment, and proposed the Bottom-Grid algorithm. The basic idea is to start from a sea-bottom triangle-grid sensor deployment, and adjust the depth of sensors to form a three-dimensional sensor deployment. The goal is to use as few sensors as possible to cover the 3D monitoring space seamlessly. In this algorithm, global information is needed for adjusting the depth of sensors, and thus the algorithm has to operate in a manner. In 2009, Akkaya *et al.* [20] proposed a Self-deployment algorithm by adjusting the depth of sensors continuously to reduce the coverage overlap between adjacent sensors so as to improve the total coverage in the monitoring space. In [21–23], the authors discussed the network restoration method. When a coverage hole appears in the network, new sensors or redundant sensors will be sent to the position to fix the hole, so as to maintain the coverage and connectivity of the network.

The second class is *non-uniform coverage requirement sensor deployment*. Sensors are non-uniformly deployed in the monitoring space according to the distribution of targets; In 2007, Aitsaadi *et al.* [24] proposed the differentiated deployment algorithm (DDA) for lake water monitoring. Sensors are deployed in the lake according to the distribution of pollutant using a mesh line representation. This algorithm utilizes a centralized optimization method and is only suitable when the environment and targets are static. In 2008, Koutsougeras *et al.* [25] presented the Self Organizing Maps (SOM) method. Attracted by events (targets), sensors can move to areas with high density of events. Though the method was not originally designed for terrestrial sensor networks, it is expected to be applicable to UWSNs as well. In 2010, Golen *et al.* [26] proposed a scheme to estimate the probability of events in each underwater subregion, and compute the number of sensors that should be allocated in each subregion. This method also suffered from the problem of centralized implementation and high computation complexity.

In non-uniform coverage requirement sensor deployments, the concept of “event” (target) is generally well defined. The objective of sensor deployment is to cover the events and make the distribution of sensors match that of the events. This kind of research is close to practice, and reflects the sparsity characteristic of UWSNs. However, existing methods fall short in the following aspects:

(1) Existing methods all reply on centralized optimization methods, and are difficult to realize in practice;
(2) Most of the methods are designed for static environments. For dynamic environments with uncertain events, these methods cannot be used to adjust the positions of sensors to guarantee desired monitoring quality;
(3) These methods have not taken into account the influence of the water flow;
(4) The performance evaluation metrics of the event-driven underwater sensor deployment are not fully quantifiable.

To address these problems, we study the problem of three-dimensional underwater sensor deployment with the non-uniform coverage requirement. Inspired by particle swarm systems, and we propose PSSD (particle swarm inspired underwater sensor deployment), a distributedly realizable underwater sensor deployment algorithm. By simulating the flying behavior of particles and introducing crowd control, PSSD can drive the sensors to positions with high density of events, and avoid over crowding simultaneously. The design and simulation of PSSD utilize the Lagrange flow model, and are evaluated using an information theoretical metric. The rest of the paper is organized as follows: in Section 2, the underwater sensor deployment problem and its performance metrics are formally defined. In Section 3, the PSSD algorithm is presented in detail. An extensive evaluation is provided in Section 4 with our conclusions in Section 5.

## 2. Preliminaries

### 2.1. Underwater Sensor Deployment Problem

Suppose an underwater monitoring space *A*. Denote a dynamic event by *e*, and the set of events as *E* = {*e*_1_,*e*_2_, ⋯,*e_m_*}, (*e_i_*∈*A*,*i*=1,2, ⋯*m*). Let the set of sensors be *S* = {*s*_1_,*s*_2_, ⋯*s_n_*}, and each sensor *s_j_* (1 ≤ *j* ≤ n) has the ability of sensing, communication and moving. Denote the attribute vector of *s_j_* as ${\text{B}}_{j}=\langle r_{j}^{s},r_{j}^{c},l_{j},{\text{p}}_{j}\rangle$, where $r_{j}^{s}\geq 0$, $r_{j}^{c}\geq 0$, *l_j_* ≥0 describe the sensing radius, communication radius, and the maximal moving range of *s_j_*, respectively and **p***_j_* is the current position of *s_j_*. All sensors have the same attributes except for position in a homogeneous network, namely, $r_{j}^{s}=r^{s}$, $r_{j}^{c}=r^{c}$, *l_j_* = *l* (1≤ *j* ≤ *n*). Sensors can detect events and communicate with their adjacent sensors to exchange information (the number of covered events). The task of sensors is to cover events and collect relevant information.

#### Definition 1

**Figure 1.**
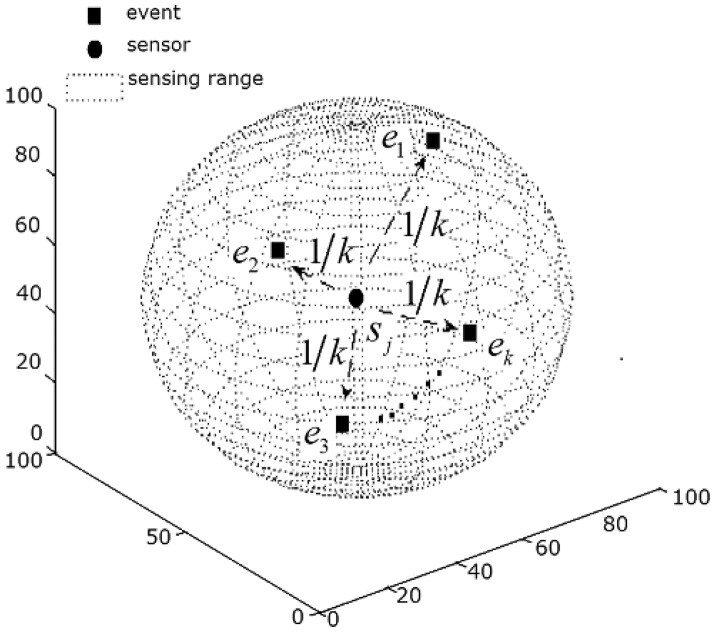
Sensor *s_j_* covering *k* events.

Coverage. Consider an event at location *e_i_* ∈ *A*, if $d\left(e_{i},s_{j}\right)\leq r_{j}^{s}$, we call *e_i_* is covered by *s_j_*. Here *d*(*e_i_*, *s_j_*) is the Euclidean distance between *e_i_* and *s_j_*. If *e*_1_,*e*_2_,…,*e_k_* are all covered by *s_j_* (Figure 1), *s_j_* is “divided” equally among all events, and each event shares 1/*k* of sensor *j*.

#### Definition 2

Coverage degree. Given a collection of sensor placement, the number of sensors shared by event *e_i_* is the coverage degree of *e_i_*. It can be computed as follows:

(1) $$D_{A}(e_{i})=\sum_{s_{j}\in S}\frac{\text{I}\left\{d\left(e_{i},s_{j}\right)\leq r_{j}^{s}\right\}}{\sum_{e_{u}\in E}\text{I}\left\{d\left(e_{u},s_{j}\right)\leq r_{j}^{s}\right\}}$$

where I(·) is an indicator function. $\underset{e_{u}\in E}{\text{∑}}\text{I}\left\{d\left(e_{u},s_{j}\right)\leq r_{j}^{s}\right\}$ is thus the number of events that sensor *s_j_* covers. The physical meaning of *D_A_*(*e_i_*) is the total shares of coverage of event *e_i_* among all sensors.

The underwater sensor deployment problem is to place sensors in the underwater monitoring space *A* to cover as many events as possible, and make the coverage degree of every event as equal as possible. In other words, the sensor distribution matches the event distribution.

### 2.2. Performance Metrics

The underwater sensor deployment problem is to place sensors to cover events, and make the coverage degree of every event as equal as possible. So, we need a metric to evaluate how equal the coverage degree of every event is.

Then, we borrow the concept of “entropy” from Information Theory. As we know, the closer the probabilities of *q* outcomes are, the larger the entropy is, and when the probabilities of *q* outcomes are equal, the entropy reaches the maximum value *logq*. So, “entropy” is suitable as the metric to evaluate how equal the coverage degree of every event is. Furthermore, because the maximum value of entropy is known, it is easy to be normalized for evaluation. Normalizing *D_A_*(*e_i_*), we have:

(2) $${D^{\prime }}_{A}\left(e_{i}\right)=\frac{D_{A}(e_{i})}{\sum_{e_{u}\in E}D_{A}(e_{u})}$$

Clearly, *D*′*_A_*(*e_i_*) ∈[0,1]. When *D_A_*(*e_i_*)=*c*, namely all events have the same normalized coverage degree, $D{'}_{A}(e_{i})=\frac{1}{m}.$

#### Definition 3

Coverage entropy of the event set. It measures the uniformity of the coverage degree of events, and can be computed as follows:

(3) $$H_{A}\left(E\right)=-\sum_{e_{i}\in E}{D^{\prime }}_{A}\left(e_{i}\right)\log{D^{\prime }}_{A}\left(e_{i}\right)$$

Clearly, *H_A_*(*E*) ≤ *m*. When the number of sensors is large, *H_A_*(*E*) remains the same when only a subset of sensors covers the events equally. Therefore, we further introduce a penalty factor associated with the percentage of sensors covering events in the final definition of coverage efficiency as follows.

#### Definition 4

Coverage efficiency of the event set. It evaluates the overall performance of sensor deployment, and is computed as:

(4) $$\eta \left(E\right)=\alpha \frac{H_{A}\left(E\right)}{\log m}+\beta \frac{\hat{n}}{n}$$

where *α*, *β* ∈[0,1], and *α* + *β* =1, *n̂* is the number of sensors covering events. In what follows, we prove some properties of coverage efficiency.

##### Lemma 1

Coverage efficiency *η*(*E*) of event set E will be maximized when all sensors cover some events and all the coverage degree of events are equal.

The proof of Lemma 1 is in Appendix.

##### Lemma 2

Coverage efficiency of the events set *η*(*E*) will increase when the number of sensors covering events is fixed, and the coverage degree of events becomes equal.

The proof of Lemma 2 is in Appendix.

From on Lemmas 1 and 2, we observe that *η*(*E*) is a suitable metric to characterize event-driven underwater sensor deployment.

### 2.3. Water Flow Field

Underwater environments are complicated and dynamic. Water flow and vortex may introduce disturbance to events and sensor measurements. We introduce the water flow field model discussed in [27] to simulate the underwater environment and test the sensor deployment algorithms. For an incompressible fluid, it can be described by a stream function *ψ*, from which the two components of the divergenceless velocity field **u** ≡ (*u*, *v*) are computed as:

(5) $$\begin{array}{cc} u=-\frac{\partial \psi }{\partial y}; & v=\frac{\partial \psi }{\partial x}, \end{array}$$

where *u* is the zonal (eastward) component of the velocity field and *v* is the meridional (northward) one. The trajectory of a Lagrange device that moves with the current is the solution of the following system of Hamiltonian ordinary differential equations:

(6) $$\begin{array}{cc} \dot{x}=-\partial_{y}\psi \left(x,y,t\right), & \dot{y}=-\partial_{x}\psi \left(x,y,t\right) \end{array}$$

The water flow jet model is given by:

(7) $$\psi \left(x,y,t\right)=-\tanh\left[\frac{y-B\left(t\right)\sin\left(k\left(x-ct\right)\right)}{\sqrt{1+k^{2}B^{2}\left(t\right)\cos^{2}\left(k\left(x-ct\right)\right)}}\right]$$

where *B*(*t*) =*Av* + *ε* cos (*ωt*). The flow induces a net mass transport along the current, and a vigorous chaotic mixing across the current in a wide range of parameters. The parameter *k* sets the number of meanders in the unit length; *c* is the phase speed with which they drift downstream; the time-dependent function *B* modulates the width of the meanders: *Av* determines the average meander width, ε is the amplitude of the modulation, and ω is its frequency. Water flow leads to the movement of events, and can also be utilized for sensors to move so as to conserve energy.

## 3. PSSD (Particle Swarm Inspired Underwater Sensor Deployment) Algorithm

In this paper, a particle swarm inspired underwater sensor deployment algorithm (PSSD) is proposed to solve the underwater sensor deployment problem. Particle swarm optimization (PSO) is a population based stochastic optimization technique developed by Eberhart and Kennedy [28,29], to simulate the social behavior of bird flocking or fish schooling. In PSO, the potential solutions are called particles. All particles have velocities that direct the flight of the particles. Particles fly across the problem space to search for the optimal position (solution). PSO is initialized with a group of random particles and then searches for the optima by following two “best” solutions to date. One is the best position that has been achieved so far by the particle itself. This is called “pbest”. The other is the best position obtained so far by any particle in the population. This is a global best and called “gbest”. In the past several years, PSO has been successfully applied in many research and application areas [30,31], and its parameters setting has been well discussed [32,33].

PSO is a centralized intelligent searching method. Inspired by the operation mechanism of particle swarm systems, we present a distributedly realizable underwater sensor deployment algorithm. Sensors correspond to the particles of PSO. Sensors moving and covering events is similar to particles searching for solutions.

Denote the number of events covered by *s_j_*:

(8) $$N_{\text{event}}\left(s_{j}\right)=\sum_{e_{k}\in E}\text{I}\left\{d\left(s_{j},e_{k}\right)\leq r_{j}^{s}\right\}$$

If an event is in the sensing range of a sensor, the sensor will detect the event and obtain its approximate location. Furthermore, the sensor will share this information with its adjacent sensors.

### Definition 5

Allowed crowd factor. Let the allowed crowd factor of *s_j_* in monitoring space *A* be:

(9) $$\delta \left(s_{j}\right)=\bar{D}\times N_{\text{event}}\left(s_{j}\right)$$

where *D̄* denotes the expected coverage degree for each event. It can be set by experience. Another reasonable way is to set *D̄* as the average value *n*/*m* for *n* sensors and *m* events.

The number of sensors in the coverage of *s_j_* is *N_near_* (*s_j_*).

The set of the adjacent sensors of *s_j_* is:

(10) $$K\left(s_{j}\right)=\left\{s_{k}|d\left(s_{j},s_{k}\right)\leq r_{j}^{c},k=1,2,\ldots ,n\right\}$$

So, the number of the adjacent sensors of *s_j_* is:

(11) $$N_{\text{neighbor}}\left(s_{j}\right)=card\left(K\left(s_{j}\right)\right)$$

Where card(.) is to compute the number of the elements in a set.

### Initialization

randomly scatter *n* sensors in the underwater monitoring space *A*. Sensor *s_j_* will execute the following operations according to the state of itself and its adjacent sensors.

- If *N_neighbor_* (*s_j_*) > 0, which means *s_j_* has some adjacent sensors, *s_j_* will follow the “gbest”.

Find the best adjacent sensor $s^{*}=\arg\underset{s_{k}\in K\left(s_{j}\right)}{\max}\left\{N_{\text{event}}\left(s_{k}\right)\right\}$.

**Figure 2.**
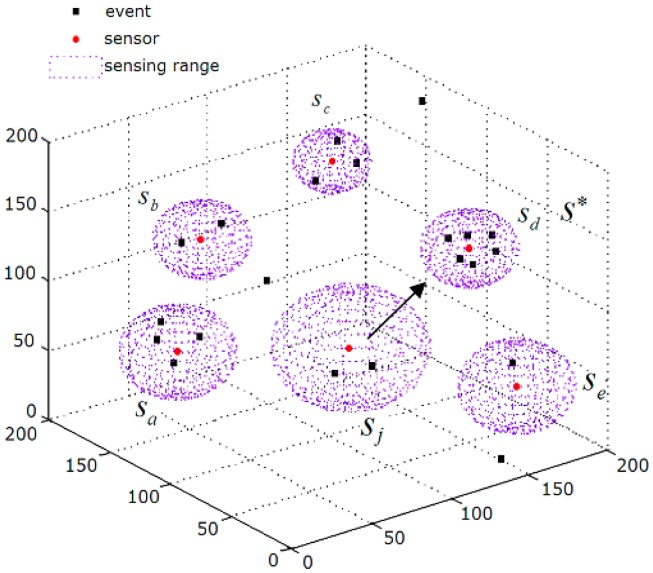
*s_j_* moves toward s* (the red points are sensors; the purple spheres represent the sensing space (coverage) of sensors; black dots are events.).

If s* is covering more events than *s_j_*, and the position of s* is not crowded, that is, *N_event_* (s*) ≥ *N_event_* (*s_j_*), and *N_near_* (s*) < *δ* (s*), then *s_j_* will move toward s*. It is depicted in Figure 2.

The expected velocity of *s_j_* is as follows:

(12) $${{\text{v}}_{\text{j}}}^{*}\left(t+1\right)={\text{x}}^{*}-{\text{x}}_{j}\left(t\right)$$

(13) $${\text{v}}_{j}^{*}\left(t+1\right)=\{\begin{array}{ll} l_{j}\frac{{\text{v}}_{j}^{*}\left(t+1\right)}{\left\|{\text{v}}_{j}^{*}\left(t+1\right)\right\|} & \text{if}\left\|{\text{v}}_{j}^{*}\left(t+1\right)\right\|>l_{j},\\ {\text{v}}_{j}^{*}\left(t+1\right) & \text{otherwise} \end{array}$$

where x* is the position of s*; **x***_j_*(*t*) is the current position of *s_j_*; *t* is the iteration number of the algorithm. The expected velocity **v**_j_* (*t* + 1) is restricted by the maximal moving range *l_j_*, and the adjustment of velocity is given in Equation (13).

**Figure 3.**
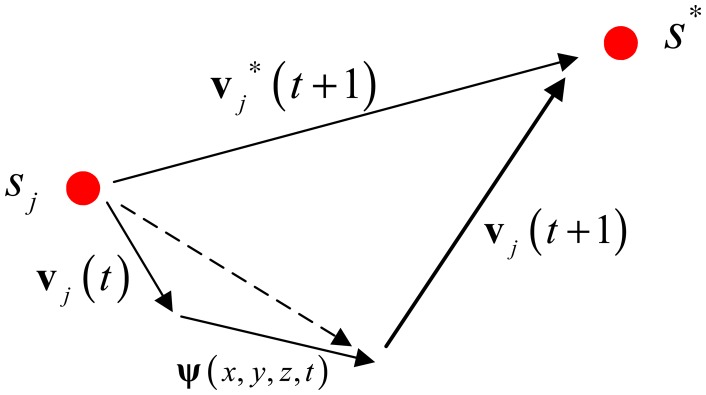
the computing for the actual velocity.

Since the movement of the sensor will be affected by its inertia and the water flow, *s_j_* should either overcome or take advantage of them in moving. Therefore, the actual velocity of *s_j_* is computed according to Equation (14) as illustrated in Figure 3.

(14) $${\text{v}}_{j}\left(t+1\right)={\text{v}}_{j}^{*}\left(t+1\right)-{\text{v}}_{j}\left(t\right)-\psi \left(x,y,z,t\right)$$

where **v***_j_*(*t*) is the current velocity of *s_j_*. It represents the sensor's inertia. **v***_j_* (*t*+1) is the actual velocity of *s_j_*. **ψ**(*x*,*y*,*z*,*t*) is the local water flow field (the water flow velocity vector can be measured by acoustic Doppler velocity measurement ADCP) detected by *s_j_*.

Then, the position of *s_j_* will be updated as follows:

(15) $${\text{x}}_{j}\left(t+1\right)={\text{x}}_{j}\left(t\right)+{\text{v}}_{j}\left(t+1\right)$$

This sensor movement strategy not only inherits the flying behavior of particle swarm, but also takes into account the influence of water flow to conserve energy.

After the movement, if *N_event_* (*s_j_*) increases, the moving behavior is successful, otherwise the sensor will move back to its original position.

II. If *N_neighbor_* (*s_j_*) =0, or equivalently, *s_j_* has no adjacent nodes, *s_j_* will follow the “pbest”. “pbest” is the best position that has been achieved so far by *s_j_* itself. It is recorded by *s_j_*.
II. (a) Find the best recorded position ${\hat{x}}_{j}$. If ${\hat{x}}_{j}$ is not the current position of *s_j_*, *i.e.*, ${\hat{x}}_{j}\neq x_{j}(t)$, *s_j_* will move toward ${\hat{x}}_{j}$. The expected velocity is computed as:
  (16) $${\text{v}}_{j}^{*}\left(t+1\right)={\hat{\text{x}}}_{j}-{\text{x}}_{j}\left(t\right)$$
  Equation (13) can also be applied to adjust the velocity. The actual velocity and the updated sensor position follow Equations (14) and (15).
II. (b) If ${\hat{x}}_{j}$ is the current position of *s_j_*, *i.e.*, ${\hat{x}}_{j}=x_{j}(t)$, *s_j_* will move randomly.

After the movement, if *N_event_* (*s_j_*) increases, the moving behavior is successful, otherwise the sensor will move back to its original position.

Based on the description above, we present the complete particle swarm inspired underwater sensor deployment algorithm in Algorithm 1.


**Algorithm 1.** Particle Swarm Inspired Underwater Sensor Deployment Algorithm.
**Input**: sensing range *r^s^*, communication range *r^c^*, the maximal moving range *l*, the maximal iteration number *I*.**Output**: the positions of sensors in the underwater monitoring space1.*S* → Randomly deploy sensors in the monitoring space;2.**for**
*step* → 1 to *I*
**do**3. *N_event_(s_j_)* →*detect1(s_j_)* /* detect the number of the events covered by s_j_ itself */;4. *N_neighbor_(s_j_)* →*detect2(s_j_)* /* detect the number of the adjacent sensors */;5. *N_near_(s_j_)* →*detect3(s_j_)* /* detect the number of the near sensors */;6. **if**
*N_neighbor_(s_j_)* > 0 **then**7.  find the best adjacent sensor s*;8.  **if**
N_event_(s*) > *N_event_(s_j_)* and N_near_(s*) < δ (s*)
**then** /* follow the gbest */;9.   move towards s*;10.  **end**11 **else** find the best position of *s_j_* during its moving (
${\hat{x}}_{j}$);12.  **if**
${\hat{x}}_{j}!=x_{j}(t)$
**then** /*follow the pbest */;13.   move towards 
${\hat{x}}_{j}$,14.  **else**15.   move randomly;16.  **end**17. **end**18.**end**


## 4. Performance Evaluation

In order to evaluate the performance of the proposed algorithm PSSD, we conduct several rounds of Monte Carlo simulations using Matlab. We define the attribute and moving strategy of the sensors in the program. Because the existing event-driven sensor deployment algorithms are all centralized optimization methods while our algorithm is realized in a distributed manner, a comparison between them is not very meaningful. However, for completeness, we still compare the SOM (self organizing maps) algorithm in [25] with PSSD. As a centralized method, SOM can utilize the global information to search for the solution, so the quality of its solution is guaranteed. In the comparison experiments, if PSSD does better than SOM, the validity of PSSD will be verified.

The parameter settings of the simulations are given in Table 1. The evaluation metrics for both algorithms include *η*(*E*) defined in Section 2.2, and the convergence speed. In *η*(*E*), *α* = *β* = 0.5.

### 4.1. Static Environments

In a 200 × 200 × 200 sea-column monitoring space, three sets of experiments are conducted:

Experiment I: 40 events are uniformly distributed. A total of six sensors are deployed.
Experiment II: 40 events are non-uniformly distributed following a T-shape. A total of six sensors are deployed.
Experiment III: 40 events are non-uniformly distributed following a line. A total of six sensors are deployed.

**Figure 4.**
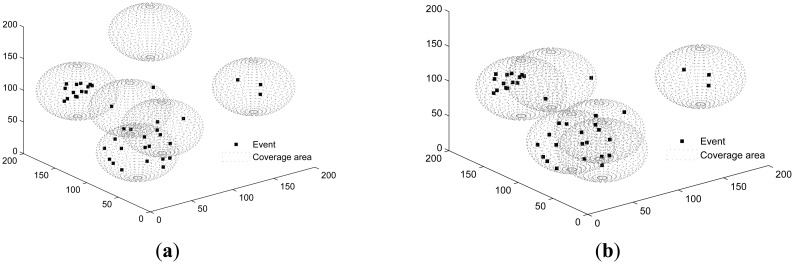
Events randomly distributed. (**a**) solution of SOM (self organizing maps); (**b**) solution of PSSD (particle swarm inspired underwater sensor deployment).

**Figure 5.**
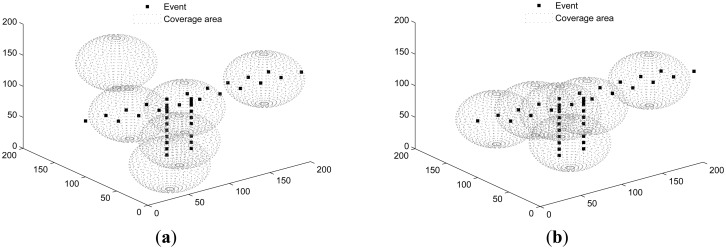
Events distributed non-uniformly in T-shape. (**a**) solution of SOM; (**b**) solution of PSSD.

**Figure 6.**
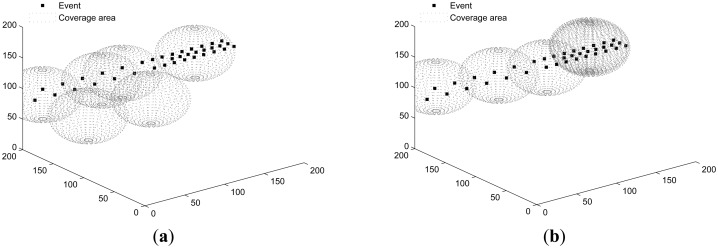
Events distributed non-uniformly along a line. (**a**) solution of SOM; (**b**) solution of PSSD.

**Figure 7.**
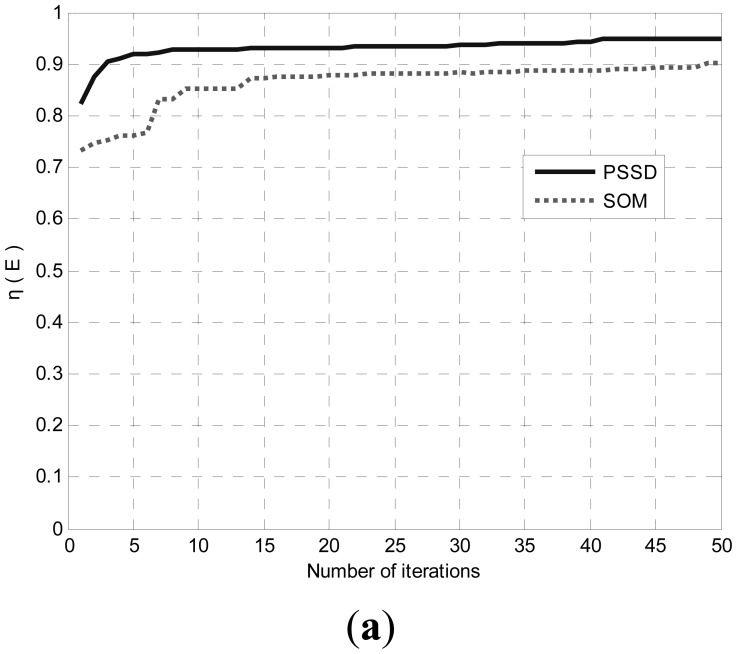

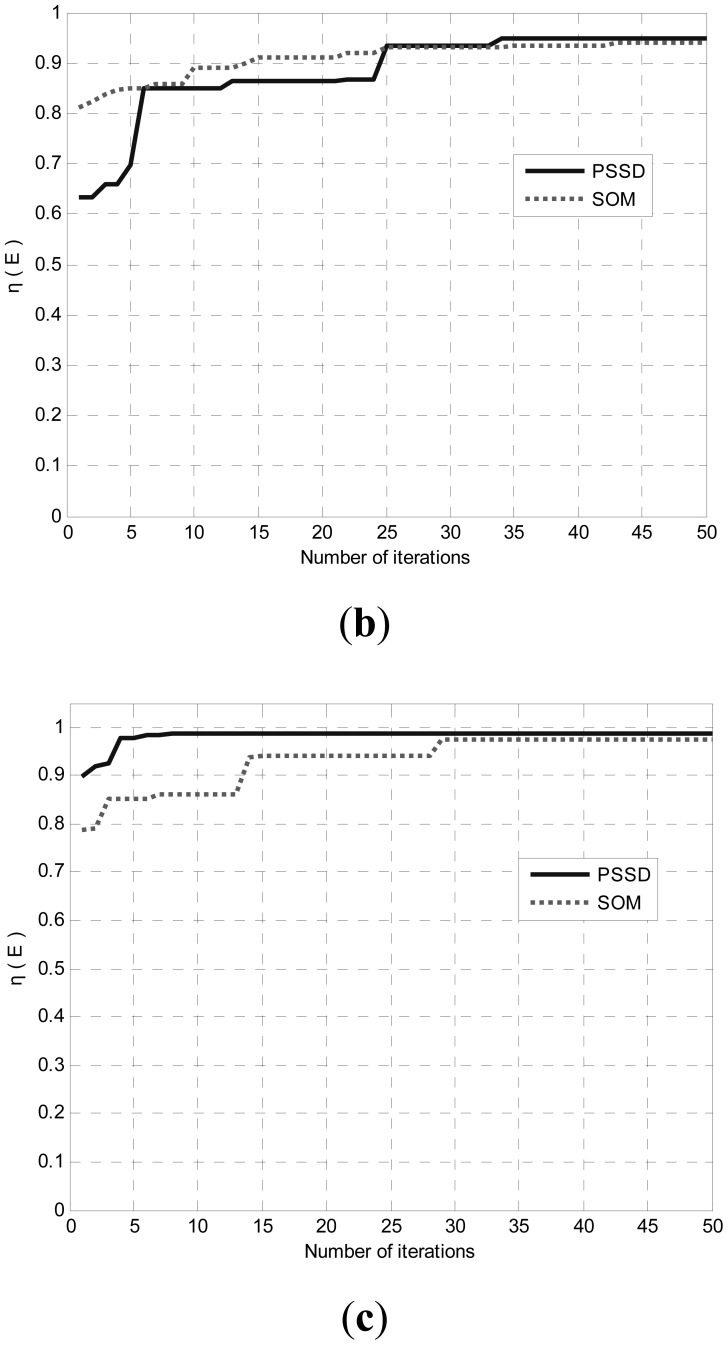
The evolving of η(*E*) of the two algorithms in three sets of experiments. (**a**) Experiment I; (**b**) Experiment II; (**c**) Experiment III.

Both SOM and PSSD are applied to all scenarios. Figures 4, 5 and 6 show the results. Black dots denote events, and the spheres represent the sensing space (coverage) of sensors. At the center of each sphere is a sensor. It is clear that with PSSD, all sensors cover some events, and the distribution of sensors matches that of events. In contrast, with SOM, a few sensors cannot cover any events, and the distribution of sensors does not match that of events. Figure 7 shows the evolution of *η*(*E*) with both algorithms in three sets of experiments. It can be seen that PSSD reaches higher coverage efficiency than SOM. Furthermore, the sensors require fewer steps to reach good position, which indicates the fast convergence speed of PSSD. Also note that PSSD is a distributedly realizable algorithm, while SOM is a centralized algorithm.

Table 2 summarized the results for static environments. In each set of experiments, PSSD algorithm runs 20 times to determine the optimal and average coverage efficiency denoted by η*(*E*) and *η̄*(*E*), respectively. The average running time is estimated as the time for each sensor to move 50 steps measured on a desktop PC with Intel Core2 CPU @ 2.00 GHz, 1G memory. Again, we observe that PSSD achieves higher coverage efficiency than SOM (1 is the best possible value) at very low computation costs. As deterministic method, SOM just runs once.

### 4.2. Dynamic Environments

**Figure 8.**
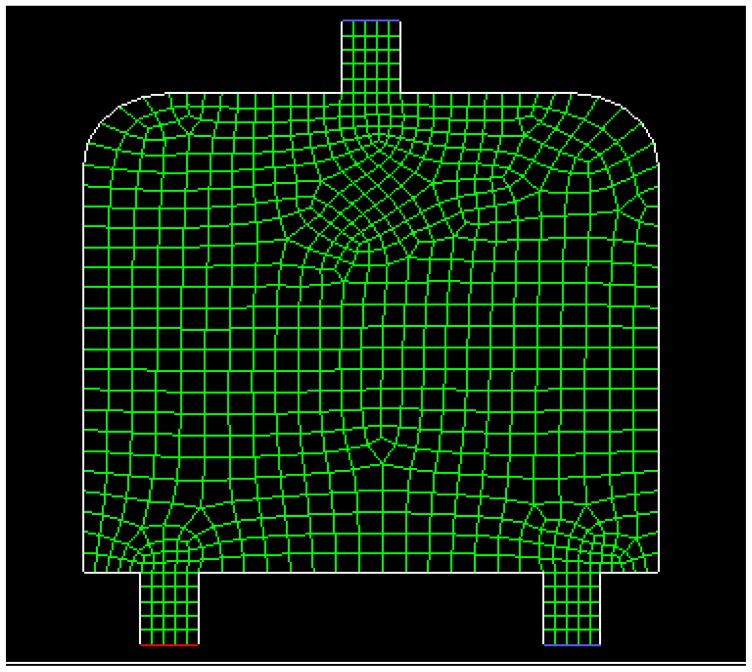
Water flow environment and established grids.

**Figure9.**
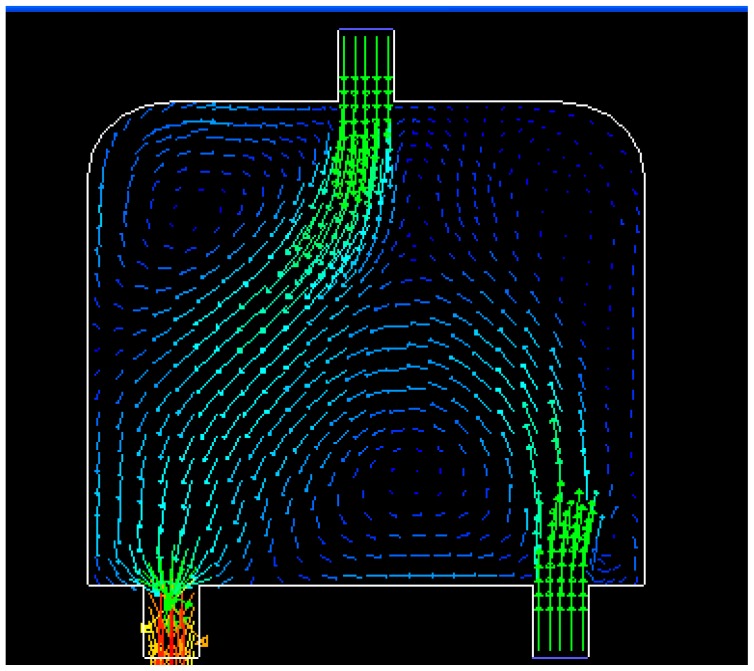
The distribution of water flow velocity.

Dynamic simulations are conducted in a special water flow environment. Figure. 8 illustrates the water flow environment and the established grids. Figure 9 depicts the distribution of water flow velocity. The water flow velocity is generated using the model described in Section 2.3, and the model parameters are given in Table 3. The update period *T* for sensors in PSSD is 1 s.

**Figure 10.**
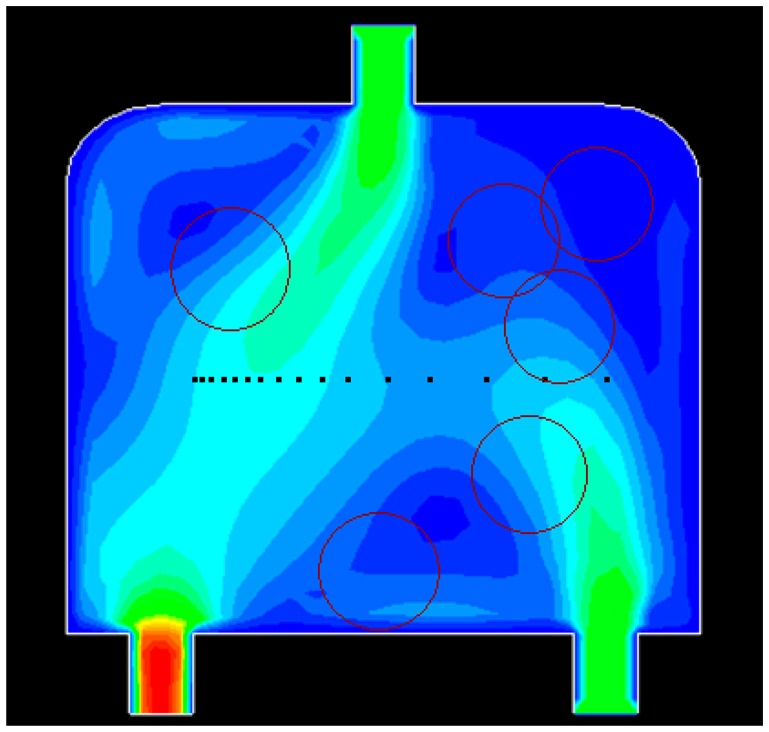
The distribution of events and sensors at t1.

**Figure 11.**
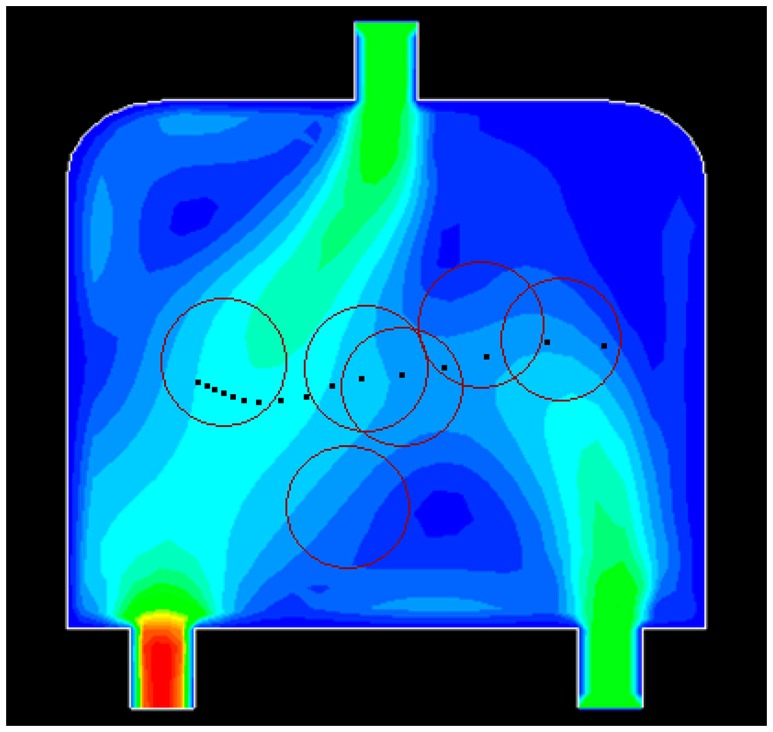
The distribution of events and sensors at t2.

**Figure 12.**
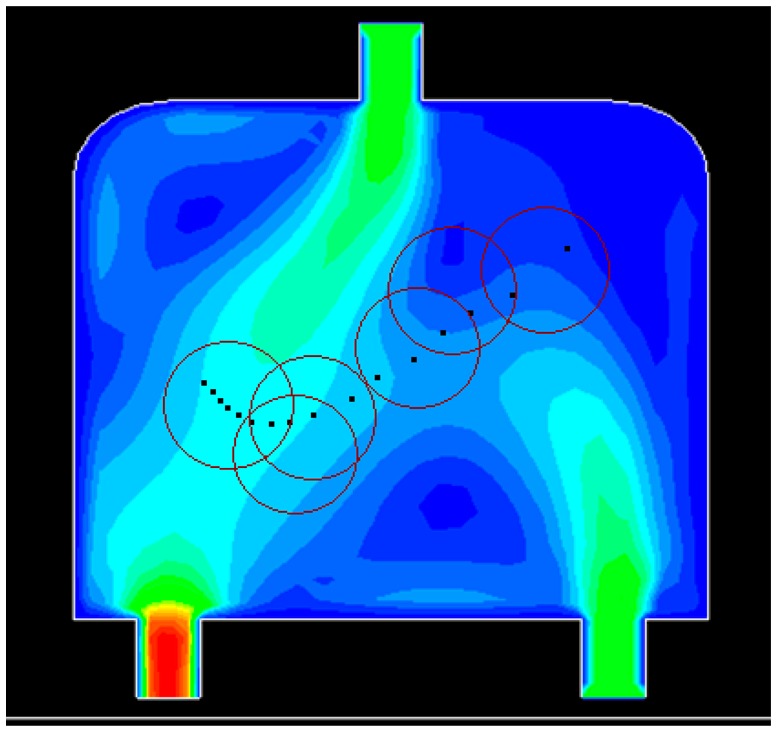
The distribution of events and sensors at t3.

**Figure 13.**
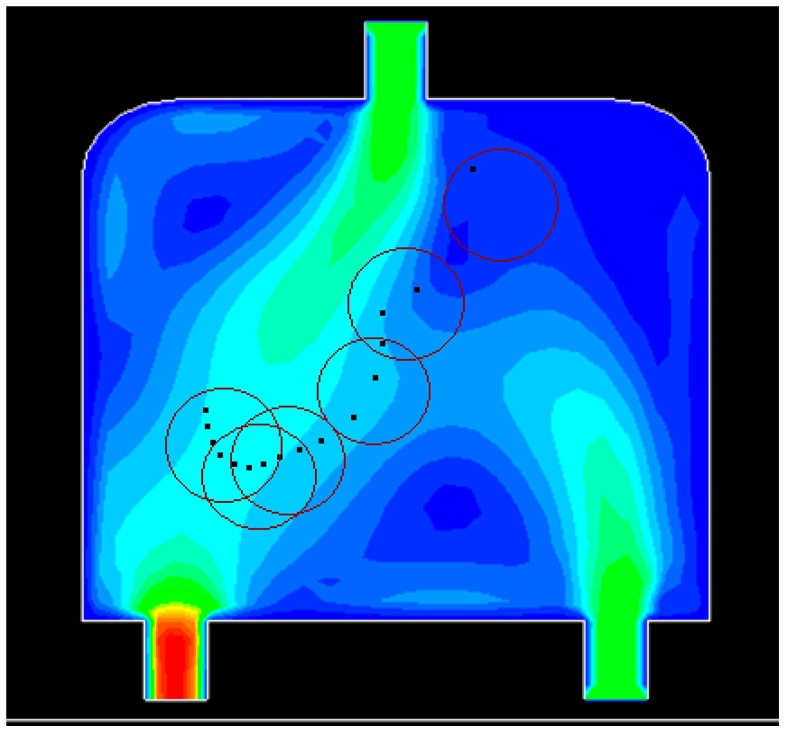
The distribution of events and sensors at t4.

**Figure 14.**
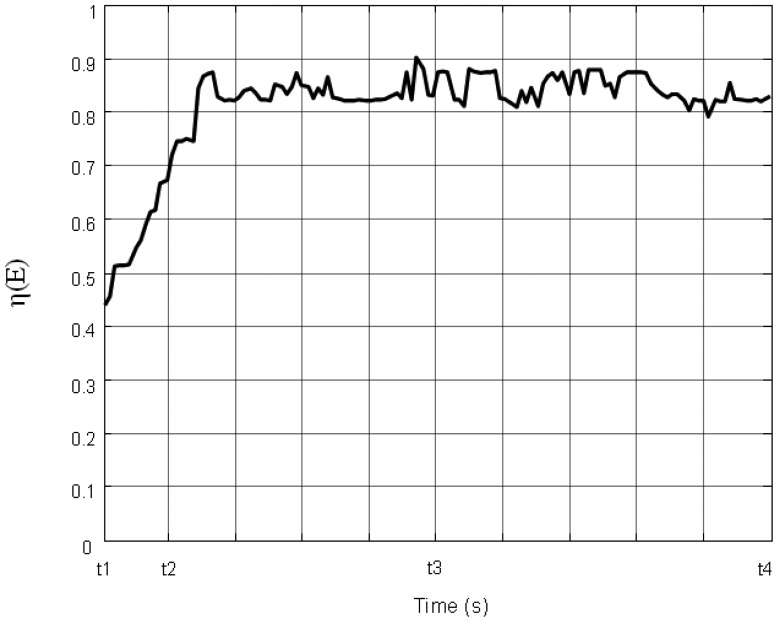
The varying of the coverage efficiency η (*E*).

Initially, 16 events are distributed non-uniformly along a line in the water flow environment (Figure 10). The black dots denote the events. Six sensors are deployed randomly in the field. The red circle represents the sensing area of a sensor. Figures 10, 11, 12 and 13 show the distribution of the events and sensors at four time points during the execution of the algorithm. Evidently, the sensors swarm toward and eventually cover the events, adjusting their positions with the moving events, and maintaining the optimal coverage over time. Figure 14 shows the changes in the coverage efficiency *η*(*E*) over time. It is clear that after time t2, the sensor deployment becomes stable with *η*(*E*) fluctuates between 0.8 and 0.9, achieving high monitoring quality.

**Figure 15.**
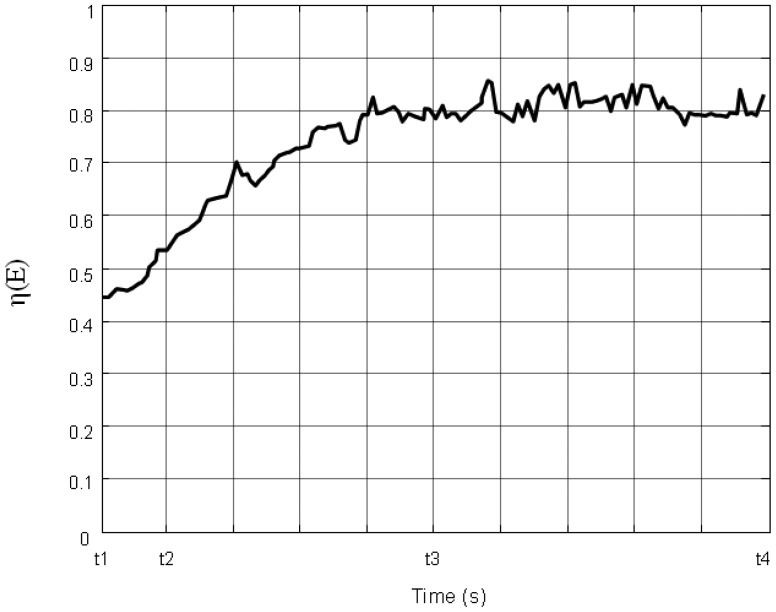
The varying of the coverage efficiency η (*E*) in the comparison experiment.

Because the proposed moving strategy can overcome or use the inertia and the water flow to move, as described in Equation (14), the moving efficiency of the sensor is improved. Furthermore, sensors can save energy. Another experiment is conducted to show this point. We modify Equation (14) as ${\text{v}}_{j}\left(t+1\right)={\text{v}}_{j}^{*}\left(t+1\right)$. The dynamic simulation experiment result is shown in Figure 15. It shows that the sensors spend more time on moving to cover the events. As a result, more energy is consumed. Even if in the stable stage, *η* (*E*) is around 0.8. The monitoring quality decreased.

**Figure 16.**
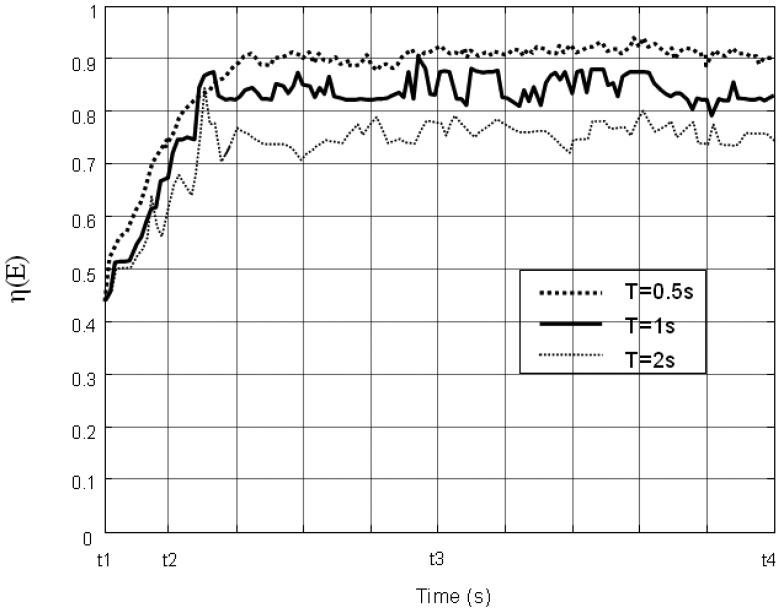
The varying of the coverage efficiency η (*E*) with different parameter *T*.

Impact of the update period: We conduct three sets of dynamic simulations with different parameters *T* (0.5 s, 1 s, and 2 s). The experimental results are shown in Figure 16. It can be seen that the smaller *T* is, the more efficiently the sensors move, and the better coverage efficiency the network achieves. However, if *T* is too small, the sensors will collect the environment information (covered events, adjacent sensors, and near sensors) more frequently, and need to update the velocity frequently. Thus, a smaller *T* leads to higher communication overhead as well as higher computing cost. An optimal choice of *T* would need to account for the velocity of the water flow, sensor mobility, communication latency and remaining energy. This will part of our future work.

## 5. Conclusions

In this paper, we investigated the problem of event-driven underwater sensor deployment:

(1) We introduced a novel performance evaluation metric, termed coverage efficiency of the events set, which not only reflects the total number of the sensors covering events, but also quantifies the matching between distributions of sensors and events.
(2) Inspired by particle swarm system, we propose PSSD, a distributedly realizable underwater sensor deployment algorithm. By simulating the flying behavior of the particles (following “gbest” and “pbest”) and introducing a crowd control factor, PSSD can drive sensors to swarm to and cover the dynamic events adaptively, and make the distribution of sensors match that of events.
(3) Simulations under both static and dynamic setting show that the algorithm can solve the underwater sensor deployment problem effectively, with fast convergence rate, and amiable to distributed implementation.

As an ongoing objective, we plan to rigorously prove the convergence of PSSD, and conduct experiments in realistic underwater environments.

## Figures and Tables

**Table 1.** Parameter settings.

**Experiment parameters**			**Algorithm parameters**	

*r^s^* (m)	*r^c^* (m)	*l* (m)	*D̄*	*I*
sensing radius	communication radius	the maximal moving range	the expected coverage degree for each event	Max number of iterations

40	80	15	1	50

**Table 2.** Summary of Results in Static Experiments.

**Experiment set**	**I: Randomly distributed**		**II: T-shape non-uniformly distributed**		**III: Line-shape non-uniformly distributed**	
algorithms	PSSD	SOM	PSSD	SOM	PSSD	SOM
the optimal coverage efficiency η*(*E*)	0.9707	0.905	0.9708	0.945	0.9860	0.976
the average coverage efficiency *η̄*(*E*)	0.9634	0.905	0.9535	0.945	0.9847	0.976
average running time (ms)	0.0921	0.024	0.1003	0.063	0.1012	0.055

**Table 3.** Parameters of the water flow field.

**Parameter**	***k***	***c***	***Av***	***ε***	***ω***
value	2*π*/7.5	0.12	1.2	0.3	0.4

## Appendix

**Lemma 1**: Coverage efficiency *η* (*E*) of event set *E* will be maximized when all sensors cover some events and all the coverage degrees of events are equal.

**Proof**: Suppose stochastic vector $\text{Y}=\frac{1}{{{\text{D}}^{\prime }}_{A}}$, that is $y_{i}=\frac{1}{D^{\prime }{}_{A}(e_{i})}.\log Y$ is a convex function on the set of positive real numbers, so:

E[logY]≤log(E[Y])

$$\sum_{i=1}^{m}{D^{\prime }}_{A}\left(e_{i}\right)\log y_{i}\leq \log\sum_{i=1}^{m}{D^{\prime }}_{A}\left(e_{i}\right)y_{i}$$

$$\sum_{i=1}^{m}{D^{\prime }}_{A}\left(e_{i}\right)\log\frac{1}{{D^{\prime }}_{A}\left(e_{i}\right)}\leq \log\sum_{i=1}^{m}{D^{\prime }}_{A}\left(e_{i}\right)\frac{1}{{D^{\prime }}_{A}\left(e_{i}\right)}=\log m$$

$$H_{A}\left(E\right)\leq \log m$$

If ${D^{\prime }}_{A}\left(e_{i}\right)=1/m$, which means all the coverage degree of events are equal:

$$H_{A}\left(E\right)=\sum_{i=1}^{m}{D^{\prime }}_{A}\left(e_{i}\right)\log m=\log m$$

Furthermore, if all sensors cover events, that is $\overset{⌢}{n}=n$, then

$$\eta (E)=\alpha \frac{H_{A}(E)}{\log m}+\beta \frac{\overset{⌢}{n}}{n}=\alpha +\beta =1(\text{the}\;\text{maximal}\;\text{value})$$

**Lemma 2**: Coverage efficiency of the events set *η* (*E*) will increase when the number of sensors covering events is fixed, and the coverage degree of events becomes equal.

**Proof**: Suppose ${D^{\prime }}_{A}\left(e_{1}\right),{D^{\prime }}_{A}\left(e_{2}\right),\cdots ,{D^{\prime }}_{A}\left(e_{m}\right)$ are the coverage degree of *e*_1_,*e*_2_,…,*e**_m_*, and $\begin{array}{ccc} {D^{\prime }}_{A}\left(e_{i}\right) & > & {D^{\prime }}_{A}\left(e_{j}\right) \end{array}$, *i*,*j* =1,…,*m*. If ${D^{\prime \prime }}_{A}\left(e_{i}\right)={D^{\prime }}_{A}\left(e_{i}\right)-ɛ$, ${D^{\prime \prime }}_{A}\left(e_{j}\right)={D^{\prime }}_{A}\left(e_{j}\right)+ɛ$, where $0<2ɛ<\left({D^{\prime }}_{A}\left(e_{i}\right)-{D^{\prime }}_{A}\left(e_{j}\right)\right)$, and the rest coverage degrees of events don't change, the increment of *η*(*E*) will be:

$$\begin{array}{ll} \eta^{\prime }\left(E\right)-\eta \left(E\right) & =\frac{\alpha }{\log m}\left({H^{\prime }}_{A}\left(E\right)-H_{A}\left(E\right)\right)\\ & =\frac{\alpha }{\log m}\{\left[-{D^{\prime \prime }}_{A}\left(e_{i}\right)\log{D^{\prime \prime }}_{A}\left(e_{i}\right)-{D^{\prime \prime }}_{A}\left(e_{j}\right)\log{D^{\prime \prime }}_{A}\left(e_{j}\right)\right]+\left[{D^{\prime }}_{A}\left(e_{i}\right)\log{D^{\prime }}_{A}\left(e_{i}\right)+{D^{\prime }}_{A}\left(e_{j}\right)\log{D^{\prime }}_{A}\left(e_{j}\right)\right]\}\\ & =\frac{\alpha }{\log m}\left\{\left[{D^{\prime }}_{A}\left(e_{i}\right)\log{D^{\prime }}_{A}\left(e_{i}\right)+{D^{\prime }}_{A}\left(e_{j}\right)\log{D^{\prime }}_{A}\left(e_{j}\right)\right]-\left[\left({D^{\prime }}_{A}\left(e_{i}\right)-ɛ\right)\log\left({D^{\prime }}_{A}\left(e_{i}\right)-ɛ\right)+\left({D^{\prime }}_{A}\left(e_{j}\right)+ɛ\right)\log\left({D^{\prime }}_{A}\left(e_{j}\right)+ɛ\right)\right]\right\} \end{array}$$

Let $f\left(x\right)=\left({D^{\prime }}_{A}\left(e_{i}\right)-x\right)\log\left({D^{\prime }}_{A}\left(e_{i}\right)-x\right)+\left({D^{\prime }}_{A}\left(e_{j}\right)+x\right)\log\left({D^{\prime }}_{A}\left(e_{j}\right)+x\right)$, then:

$$\eta^{\prime }\left(E\right)-\eta \left(E\right)=\frac{\alpha }{\log m}\left(f\left(0\right)-f\left(ɛ\right)\right)$$

$$f^{\prime }\left(x\right)=\log\frac{{D^{\prime }}_{A}\left(e_{j}\right)+x}{{D^{\prime }}_{A}\left(e_{i}\right)-x},$$

when $x\in \left(0,\frac{{D^{\prime }}_{A}\left(e_{i}\right)-{D^{\prime }}_{A}\left(e_{j}\right)}{2}\right)$, *f*′(*x*) < 0, which means *f*(*x*) is a decreasing function in this interval, then:

$$\eta^{\prime }\left(E\right)-\eta \left(E\right)=\frac{\alpha }{\log m}\left(f\left(0\right)-f\left(ɛ\right)\right)>0$$

It indicates that *η*(*E*) is increased when the coverage degree of events becomes equal.

From Lemmas 1 and 2, we observe that *η*(*E*) is a suitable metric to characterize event-driven underwater sensor deployment.
